# Gracilis muscle flap combined with a laparoscopic transabdominal approach is effective in the treatment of post-prostatectomy rectourethral fistula: A case report

**DOI:** 10.1016/j.ijscr.2022.106856

**Published:** 2022-02-25

**Authors:** Tomohiro Takeda, Tatsuya Shonaka, Chikayoshi Tani, Toshihiko Hayashi, Hidehiro Kakizaki, Yasuo Sumi

**Affiliations:** a Division of Gastrointestinal Surgery, Department of Surgery, Asahikawa Medical University, 2-1, Midorigaoka-Higashi, Asahikawa 078-8510, Japan; b Department of Plastic and Reconstructive Surgery, Asahikawa Medical University, 2-1, Midorigaoka-Higashi, Asahikawa 078-8510, Japan; c Department of Renal and Urologic surgery, Asahikawa Medical University, 2-1, Midorigaoka-Higashi, Asahikawa 078-8510, Japan

**Keywords:** RUF, rectourethral fistula, Rectourethral fistula, Prostatectomy, Gracilis muscle flap, Transperineal, Transabdominal, Laparoscopic

## Abstract

**Introduction:**

Rectourethral fistula (RUF) after prostatectomy is a rare complication; however, when it occurs it is likely to be intractable and treatment requires surgical closure of the fistula. Several approaches to fistula closure have been reported, but there is no established treatment.

**Case presentation:**

The patient was a 66-year-old man who had undergone robot-assisted laparoscopic radical prostatectomy for prostate cancer. On the 16th postoperative day, RUF was diagnosed. Cystostomy, laparoscopic ileostomy and transanal fistula closure were performed, and conservative treatment was continued for 5 months; however, the RUF remained, so the patient underwent fistula closure with a gracilis muscle flap using both transperineal and laparoscopic manipulation. Because it was a high fistula, the RUF was difficult to fill with a transperineal approach alone; however, in combination with laparoscopic manipulation, the appropriate filling of the fistula was possible.

**Clinical discussion:**

Although few reports have described the use of the laparoscopic transabdominal approach in combination with a transperineal gracilis muscle flap, the advantages of this technique are that the superior part of the fistula can be dissected, the flap can be filled more securely than with a transperineal approach alone, and transabdominal manipulation can be performed in a less invasive manner. In addition, by coordinating perineal and laparoscopic manipulation, we were able to close the fistula without organ damage by safe dissection.

**Conclusion:**

The laparoscopic approach is useful for RUF closure because it allows the interposition of the flap to reliably fill the space between the bladder and the rectum.

## Highlights

• The rectourethral fistula is a rare complication of total prostatectomy, but when it becomes intractable, it is difficult to treat. Surgical treatment is required, but there is no standard technique.
• We used a laparoscopic approach in combination with gracilis muscle flap filling via a transperineal approach.
• To our knowledge, this technique has only been reported in only one other case in the Japanese literature.
• In a case of high rectourethral fistula, the gracilis muscle flap with a laparoscopic approach allowed for safe and secure flap filling.

## 1 Introduction and importance

Most cases of iatrogenic rectourethral fistula (RUF) are caused by radical prostatectomy; the reported incidence is approximately 0.5% [1]. Rectal injury is the most common cause of RUF, but if the injury is noticed intraoperatively, RUF can often be prevented by suture repair and fasting [2], [3]. If a rectal injury is not noticed intraoperatively, RUF may be diagnosed several days to weeks after surgery by pneumaturia, the presence of fecal matter or recurrent urinary tract infections. In this case, immediate repair is difficult; thus, conservative treatment is chosen, but it is likely to be refractory [4]. Treatment requires surgical closure of the fistula. Several approaches to fistula closure have been reported, but no consensus has been achieved concerning guidelines. We herein report a case of post-prostatectomy RUF that was treated with a transperineal gracilis muscle flap using a laparoscopic transabdominal approach. This case has been reported in line with the SCARE 2020 criteria [5].

## 2 Case presentation

**Fig. 1:**
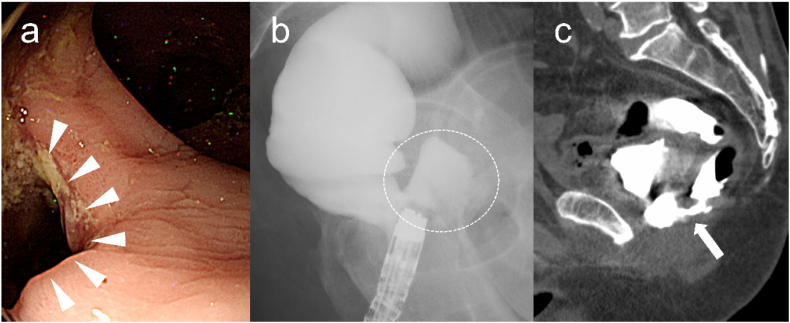
Examination findings of the patient who complained of fecal discharge from the urethra after robot-assisted laparoscopic radical prostatectomy. a) Colonoscopy: A large perforation of 2 cm in size (arrowhead) was found in the anterior wall of the lower rectum. b) Enterography: Leakage and accumulation of contrast medium on the ventral side of the rectum (dotted circle). c) CT: Contrast media traffic between the rectum and the urethra (arrow).

**Fig. 2:**
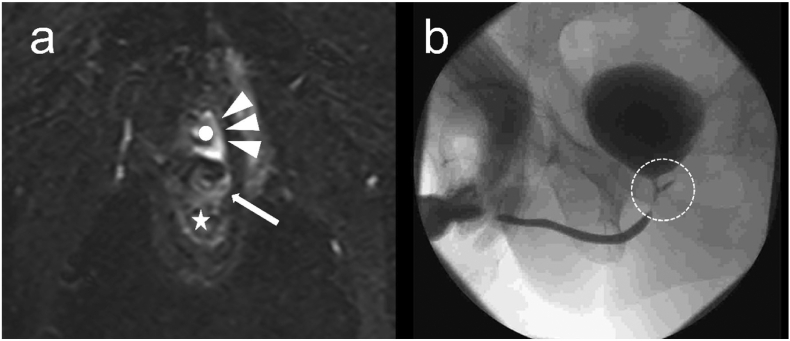
Examination findings at 5 months after cystostomy and ileostomy for RUF. a) MRI: A fistula (arrow) and fluid accumulation around the urethra (arrowhead) were observed between the rectum (☆) and urethra (○). b) Cystography: Contrast leakage was observed on the dorsal side of the urethra (dotted circle).

**Fig. 3:**
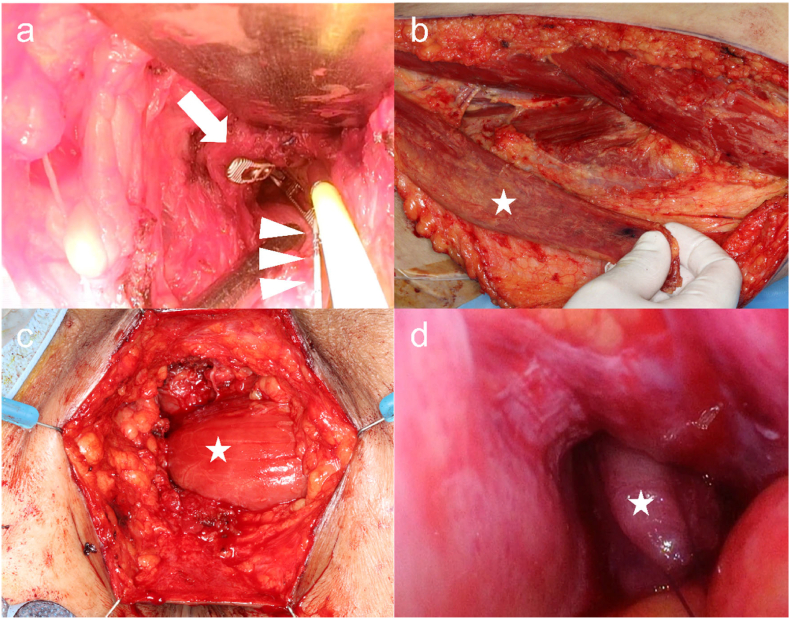
Surgical findings. a) A laparoscopic forceps (arrow) was used to access the abdominal cavity in the perineal wound, and a catheter was passed through the fistula as a landmark (arrowhead). b) A gracilis muscle flap was harvested from the left thigh. c) After closure of the fistula, the gracilis muscle flap harvested from the left thigh was inserted subcutaneously through the perineal wound. d) The gracilis muscle flap was pulled by laparoscopic manipulation, fully filling the fistula, and sutured to the peritoneum. ☆: gracilis muscle flap.

**Fig. 4:**
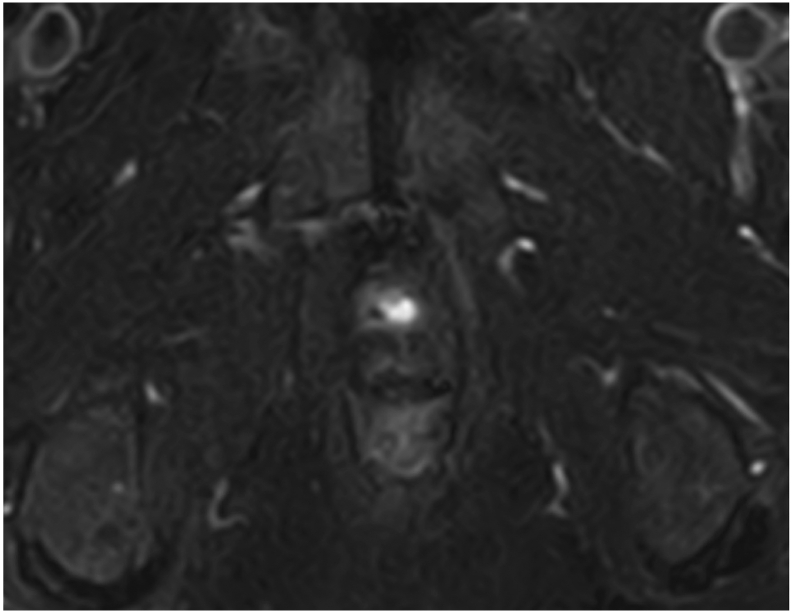
MRI at 6 months after closure of the fistula showed that the fistula had disappeared.

The patient was a 66-year-old man who had undergone robotic-assisted laparoscopic radical prostatectomy plus extended lymph node dissection for prostate cancer. The pathological results were status post endocrine therapy with residual viable acinar adenocarcinoma, ypT3bN1M0, ypStage IV. The patient was discharged from the hospital on the 14th day after surgery, but 2 days after discharge, he complained of fecal discharge from the urethra. Colonoscopy revealed a large perforation (size: approximately 2 cm) in the anterior wall of the lower rectum (Fig. 1a), and enterography and computed tomography (CT) revealed an abscess cavity (size: approximately 3 × 3 × 2.5 cm) between the rectum and urethra (Fig. 1b, c). The patient was admitted on the same day with a diagnosis of RUF and started on antimicrobial therapy. As the patient had a stable general condition, no abscess drainage was performed. The patient underwent cystostomy, laparoscopic ileostomy and transanal fistula closure on the 4th day after admission and was discharged on the 26th day after surgery. The patient continued conservative treatment for 5 months after discharge, but the RUF remained (Fig. 2a, b). A collaborative operation by urological, gastrointestinal, and plastic surgery was performed to close the fistula. A catheter (3 Fr) was inserted into the fistula using a cystoscope, which was used as a marker for fistula resection. A transverse incision was made through the perineum, and dissection of the recto-urethral space proceeded. Since it was difficult to reach the fistula through the perineum alone, we used a laparoscopic transabdominal approach to dissect the superior part of the fistula. Therefore, it was dissected using a laparoscope in combination with transabdominal manipulation. The fistula was reliably resected by combining the abdominal and perineal dissection (Fig. 3a). The pneumoperitoneum was maintained after connecting to the perineal wound, and the laparoscopic operation was not affected. The fistula was resected from the perineal wound using a catheter as a landmark, and both the rectal and urethral fistulas were closed with nodal sutures using 3-0 vicryl®. A left gracilis muscle flap was then harvested (Fig. 3b) and inserted subcutaneously into the perineal wound (Fig. 3c). It was then tractioned into the abdominal cavity by laparoscopic manipulation and fixed to the peritoneum of the bladder after confirming that the area in which the fistula was resected had been sufficiently filled (Fig. 3d). The patient was discharged on postoperative day 19. At two months after fistula closure, the cystostomy was removed. At four months after fistula closure, the closure of the fistula was confirmed by cystoscopy and colonoscopy, and ileostomy closure was performed. Six months have passed since the closure of the fistula, but the fistula has disappeared on MRI and there is no evidence of recurrent symptoms (Fig. 4). The patient will be followed up carefully with attention to urethral stricture and stenosis.

## 3 Clinical discussion

RUF is classified as complicated RUF if any one of the following is present: large perforation (>2 cm), occurrence after local therapy (e.g., radiotherapy), or severe urethral stricture. Despite a large array of approaches having been described in the literature, four approaches are most frequently used in large-volume reconstructive urology centers: transperineal, transsphincteric (York–Mason), transanal and transabdominal (open, laparoscopic, or robotic) [1]. The recommended treatment strategy for complicated RUF is flap filling via a transperineal or transabdominal approach if there is no improvement with urinary and fecal diversion [1]. This is because it is important to prevent mucosal contact between the rectum and urethra with the flap and to adequately fill with tissue with rich blood flow to prevent relapse of complicated RUF [6]. According to Harris et al., in their study of 210 RUF patients secondary to prostate cancer treatment, a transperineal approach was used in 79% of patients. A muscle flap and omentum were used in 91.9% of cases. The overall success rate was 92.8%, and the authors suggested surgical repair using a muscle or omentum flap to avoid permanent urinary diversion [7].

In a recent review, a transperineal approach using a gracilis muscle flap is recommended for complex RUF [1], [8]. The laparoscopic and transperineal approach for the treatment of RUF has some advantages over the transperineal approach alone due to better exposure to the urethra and rectum as well as easy access to the distal urethra. In addition, this access facilitates the performance of simultaneous repair of urethral pathologies, such as urethral stricture [9], [10]. Various interposition flaps can be used with the transperineal approach, such as the gracilis muscle, pediculated dartos muscle, scrotal myocutaneous, levator ani muscle, gluteus maximus, or buccal mucosa [9]. Overall, the success rates for the transperineal technique range between 75% and 100% with different types of flaps [6]. The reasons for the use of a gracilis muscle flap include accelerated healing and resistance to infection due to abundant blood flow, the ability to apply the technique in patients of any age, and good mobility [9]. Heckenbleiker et al. reported gracilis flap success rates of 91% and a frequency of use of 75% [10]. However, the narrow operative field of the transperineal gracilis muscle flap filling technique makes insertion of the flap difficult, and inadequate coverage is a problem. Fistulas involving the bladder or prostate are classified as high to intermediate in the classification of imperforate anus [11], [12]. According to the classification, post-prostatectomy RUF is considered to be a high fistula. Therefore, it is difficult to close the fistula using a transperineal approach alone, even when using a gracilis muscle flap. In the surgical technique of this case, it was difficult to fill the gracilis muscle flap through the perineum alone.

A transabdominal approach using a omentum flap could have been a treatment option [1]. However, this would require a large laparotomy and deep pelvic dissection, which is highly invasive, and identification of the fistula would also have been difficult [13]. A laparoscopic transabdominal approach is less invasive but requires a high of skill to form an omentum flap and fix it in depth [9].

We therefore decided to combine a transabdominal approach with laparoscopic manipulation. We were able to sufficiently dissect the superior part of the fistula via a laparoscopic transabdominal approach in coordination with perineal manipulation. In addition, it was possible to tow the gracilis muscle flap inserted through the perineum by laparoscopic manipulation, and it was possible to fill the high fistula. Laparoscopic manipulation in the deep pelvic region is generally more difficult. In the present case, the pelvic floor was scarred with inflammation, and careful manipulation was required. By coordinating with perineal manipulation, it was possible to dissect around the fistula without damaging vital organs. The flap could also be towed from the perineum and fixed to the cephalad peritoneum with sufficient space. These procedures could be completed in a minimally invasive operation.

There has been only one report in which laparoscopic transabdominal manipulation was used in combination with a transperineal gracilis muscle flap. In that case, it was possible to dissect the superior part of the fistula, to fill the flap more securely than with a transperineal approach alone, and to perform transabdominal manipulation with low invasiveness [14]. The same advantages were thought to have been present in this case. Of course, it should be noted that deep manipulation by laparoscopy requires a high degree of skill.

Transabdominal manipulation using a laparoscope in combination with perineal manipulation may have been effective in avoiding damage to organs such as the urethra, bladder, and rectum. Laparoscopic manipulation in the deep pelvis is difficult. In addition, male sex, a narrow pelvis, and obesity have been reported to be factors that increase the difficulty of this approach [15]. The preceding dissection by perineal manipulation made it possible to coordinate perineal and laparoscopic manipulation, as the urethra, bladder, and rectum can be secured by the transperineal approach. In addition, by applying pressure or vibration to a safe area from the perineum, even in the deep pelvic area, the safe and secure surgery can be performed by surgeons sharing information from the perineal and abdominal views with each other. Furthermore, sharing laparoscope monitor images obtained via the transperineal approach may have further enhanced coordination. Therefore, dissection was able to be safely performed from both sides via the transperineal and transabdominal approaches, and fistula closure was achieved without organ damage.

## 4 Conclusion

A transperineal approach with gracilis muscle flap is an effective technique for the treatment of RUF after radical prostatectomy because the flap can be filled into the fistula excision site securely and safely using a laparoscopic transabdominal approach.

## Provenance and peer review

Not commissioned, externally peer-reviewed.

## Funding

This research did not receive any specific grant from funding agencies in the public, commercial, or not-for-profit sectors.

## Ethical approval

Not applicable.

## Consent

Written informed consent was obtained from the patient for publication of this case report and accompanying images. A copy of the written consent is available for review by the Editor-in-Chief of this journal on request.

## CRediT authorship contribution statement

Tomohiro Takeda: writing the paper.

Tatsuya Shonaka: writing the paper/supervision.

Chikayoshi Tani: surgeon/supervision.

Hidehiro Kakizaki: surgeon/supervision.

Toshihiko Hayashi: surgeon/supervision.

Yasuo Sumi: surgeon/supervision.

## Research registration number

This case report is not “First in Man” study.

## Guarantor

Tomohiro Takeda.

## Declaration of competing interest

The authors declare no competing interests in association with the present study.
